# Nutrition and Cardiovascular Risk Factors in Four Age Groups of Female Individuals: The PEP Family Heart Study

**Published:** 2010

**Authors:** Peter Schwandt, Gerda-Maria Haas, Thomas Bertsch

**Affiliations:** 1 Arteriosklerose Praeventions Institut Munich Nuremberg, Germany; 2 Inst. Clinical Chem. Klinikum Nuremberg, Germany; 3 Ludwig Maximilians University Munich, Germany

**Keywords:** Women, Age groups, Cardiovascular risk factors, Nutrition

## Abstract

**Objectives:**

Assessment of nutritional habits and associations with cardio-metabolic risk factors in four age groups of women participating in the Prevention Education Program, Family Heart Study.

**Methods:**

Anthropometric variables, systolic and diastolic blood pressures (SBP, DBP), lipoproteins, glucose and insulin were measured in 141 children, 211 adolescents, 151 women <55 years and 150 women ≥ 55 years. Nutritional data were assessed by 7 days weighted dietary records. For statistics, SPSS 15.0 was used; associations were calculated by multiple logistic regression; p<0.05 was considered significant.

**Results:**

The prevalence of CVD risk factors was similar in children and adolescents except for hypertriglyceridemia which was >3 times more common in adolescents. Thirty six percent of junior women were overweight (BMI ≥25 kg/m^2^) and 21% had central adiposity obese. Sixty eight year-old women had a far more adverse risk profile than 35 year-old women. In terms of energy consumption, 14 year-old women had the lowest fat intake and the highest consumption of carbohydrates whereas intake of protein was lowest in 10 year-old girls. Intake of unsaturated fat was lower in youths than in adults amounting to 37 g unsaturated fat respectively 53.4% of total fat consumption. The association between energy consumption and overweight was significant and calorie intake was associated with clustering of ≥3 cardiovascular risk factors (OR: 4.72; 95% CI 1.22-18.33).

**Conclusions:**

The prevalence of CVD risk factors increased continuously from girls and adolescents to junior and senior women. However, dietary intake was different in the four age groups. Caloric intake was associated with overweight and clustering of risk factors in adult women.

## INTRODUCTION

Each 5% increase in energy intake from saturated fat is associated with a 17% increase in coronary disease in women.^1^ Diets using unsaturated fats, whole grains and an abundance of vegetables and fruits are protective against cardiovascular diseases (CVD).^2^ This is supported by a large meta-analysis of randomized controlled trials using the Bradford Hill guidelines for causality demonstrating a valid association of a limited number of dietary factors and dietary patterns with coronary heart disease (CHD).^3^ However, a meta-analysis of prospective epidemiologic studies found no evidence that saturated fat is associated with increased CVD risk; and replacement of saturated fat by unsaturated fat lowers both low density lipoprotein cholesterol (LDL-C) and high density lipoprotein-cholesterol (HDL-C) and replacement with high-carbohydrate diets increased triglycerides and decreased HDL-C.^4^,^5^ The FIT Heart study reported that adult family members with non-optimal LDL-C significantly improved their diet score.^6^ A low-fat (32% of energy) polyunsaturated fatty acid-rich (10.1% of energy) diet lowered very low density lipoprotein (VLDL) and LDL concentrations without affecting HDL-C levels in women.^7^ Adherence to lifestyle recommendations including dietary advice was associated with lower risk of CHD in 30 to 55 year-old women participating in the Nurses Heart Study.^8^ The Women’s Health Initiative describes that dietary reduction of total fat intake and increased consumption of vegetables, fruits and grains did not significantly reduce risk of CVD in postmenopausal women.^9^ With advancing age, prediction of CHD by lipids seems to decline.^10^ These data on the relation between nutrition and CVD risk factors are heterogeneous regarding dietary components, risk factors, age and gender. Therefore, we examined the prevalence of three major risk factors, i.e., hypertension, dyslipoproteinemia and adiposity, and their associations with dietary components in four age groups of women.

## METHODS

### Study population

Prevention Education Program (PEP) Family Heart Study is a prospective community-based study consisting of 15 cross-sectional surveys of CVD risk factors and lifestyle behaviors in families living in Nuremberg, Germany.^11^,^12^ PEP was approved by the ethical committee of the medical faculty of Ludwig Maximilian University of Munich, the Bavarian Ministry of Science and Education, and the local school authorities. Written informed consent was obtained from all parents together with additional oral consent from children and adolescents. The present cross-sectional study selected 653 German women participants in the 14^th^ survey of the PEP Family Heart Study. Exclusion criteria were apparent cardiovascular, metabolic, endocrine, or malignant disease, currently taken medication, non-German ethnicity to avoid ethnic bias and incomplete data sets. The four age groups included 141 girls, aged 3-11 years (mean age 9.5 ± 1.7 years), 211 female adolescents aged 12-18 years (mean age 14.7 ± 2.0), 151 women aged 19-55 years (mean age 31.4 ± 7.7) and 150 senior women above 55 years (mean age 68.6 ± 4.8) who were recruited in 2007/2008.

### Physical Examination

All measurements were made in accordance with the study manual by regularly trained research assistants performing all assessments as previously described.^13^ Waist circumference (WC) was measured to the nearest 0.1 cm according to WHO recommendations^14^ at the midpoint between the lowest rib and the iliac crest. Hip circumference (HC) was measured over the major trochanters to the nearest 0.1 cm. Two measurements were obtained, and the mean value was used in the calculation of the waist-to-height ratio (WHtR) and waist-to-hip ratio (WHR). The skinfold thickness (SFT) was measured to the nearest 0.1 mm on the left side of the body as previously described.^15^ All SFT measurements were done in triplicate and mean values were used for analysis. Systolic (SBP) and diastolic blood pressure (DBP) were measured in a sitting position.^13^ Age and gender-specific percentile curves were used for BMI^16^ and blood pressure.^17^

### Laboratory Methods

Venous blood was taken after an overnight fast. The samples were collected at central school buildings using cool boxes before centrifugation. Aliquots were stored either at -80°C for later measurements or at 4°C for lipid measurements within the following 3-4 days. Lipids and lipoproteins were measured as previously described.^13^ Fasting plasma glucose (FPG) was determined with a glucose analyzer (AU 2700 Clinical Chemistry System, Olympus, Hamburg, Germany). Apolipoproteins A-I and B were determined with a nephelometric assay (Beckman coulter on the Image 800 immunochemistry system, Beckman Coulter, Krefeld, Germany). Plasma insulin was measured on the Centaur Immunoassay System (Siemens Healthcare Diagnostics, Eschborn, Germany). Insulin resistance was determined by homeostatic model assessment^18^ and calculated as the product of the fasting plasma insulin level (in micro units per milliliter) and fasting glucose level (in millimole per liter), divided by 22.5 (HOMA Calculator version 2.2 Diabetes Trial Unit, Oxford Centre for Diabetes, Endocrinology and Medicine, Oxford, United Kingdom).

CVD risk factors were defined as described previously.^13^ Fasting hyperglycemia was characterized by FPG ≥ 100 mg/dl. Insulin resistance was defined by HOMA-IR ≥2. Central adiposity was characterized by BMI ≥ 90^th^ percentile in children and WC ≥ 88 cm in adults. General obesity was defined as BMI ≥ 90^th^ percentile in children and BMI ≥ 30 kg/m^2^ in adults.

### Nutrition

All participants were trained to precisely document their daily intake of food and beverages over 7 consecutive days including a weekend. Each participant was issued a dietary record form together with an accurately calibrated digital food scale (Soehnle Combi Plus, Nassau, Germany). The weighted dietary records of children were completed by mothers or fathers and adolescents were trained to write their own protocol under parental supervision. Lowcalorie soft drinks included all low-calorie, no added sugar, and sugar-free types of concentrated, carbonated, and ready-to-drink soft drinks. The completed records were analyzed by trained dieticians with the computer program PRODI (version 4.5, NutriScience, Freiburg, Germany) which includes “Deutscher Lebensmittelschlüssel” that was supplemented with individual special items. The PRODI data were transferred by ASCI into SPSS (version 15.0 for windows) for documentation and calculation. “Normal nutrition” was defined according to the reference values for nutrient intake in Germany, Austria and Switzerland.^19^

### Statistical Analysis

Risk factors among children and parents were described by mean ± SD, range for continuous variables and percentage for categorical variables. Pearson and Spearman correlation coefficients were calculated to assess the degree of significance. For the calculation of associations between CVD risk factors and macronutrients, multivariate logistic regression was used. All tests were 2-sided and p-values less than 0.05 were considered statistically significant. All of the statistical analyses were carried out using SPSS 15.0 version for windows (SPSS Inc., Chicago, Illinois) according to a predefined analysis plan and program.

## RESULTS

Except WHtR and WHR, all anthropometric parameters as well as lipids and apolipoproteins continuously increased over the 4 age groups whereas fasting insulin and HOMA-IR values were higher in adolescents (Table 1). The prevalence of CVD risk factors was similar in children and adolescents except for hypertriglyceridemia which was > 3 times more common in adolescents (Table 2). The prevalence of general (BMI ≥ 90^th^ percentile) and central (WC ≥ 90^th^ percentile) overweight was similar in girls and adolescents (~10%). Thirty six percent of junior women were overweight (BMI ≥ 25 kg/m^2^) and 21% were centrally obese (WC ≥ 88 cm). However, younger women had a lower prevalence of general (36%) and central (20%) overweight than senior women (49% and 53%, respectively). Among adults, 68 year-old women had a far more adverse risk profile than 35 year-old women though the prevalence of low HDL-C was twice in the younger women. Alcohol consumption of children and adolescents mainly came from soft drinks.

**Table 1:** Characteristics in four age groups of 653 female individuals (mean ± SD)

Age groups	3 – 11 years	12 – 18 years	19 – 55 years	> 55 years
Mean age, years	9.5 ± 1.7	14.7 ± 2.0	31.4 ± 7.7	68.6 ± 4.8
Median age, years	10	14	35	68
Weight, kg	35.5 ± 9.4	55.4 ± 10.8	66.9 ± 14.8	68.0 ± 10.4
Height, cm	141.3 ± 12.8	163.2 ± 7.6	165.2 ± 6.6	163.5 ± 5.7
Body mass index, kg/m^2^	17.5 ± 2.6	20.7 ±3.4	24.5 ± 4.8	25.4 ± 3.7
Waist circumference, cm	63.1 ± 7.9	73.0 ± 8.4	82.3 ± 12.0	90.5 ± 10.6
Waist to height ratio	0.5 ± 0.04	0.5 ± 0.1	0.5 ± 0.1	0.6 ± 0.1
Hip circumference, cm	74.6 ± 8.6	91.8 ± 8.5	100.7 ± 9.7	x
Waist to hip ratio	0.9 ± 0.1	0.8 ± 0.1	0.8 ± 0.1	x
Biceps, mm	5.9 ± 2.5	6.7 ± 2.8	9.1 ± 6.0	x
Triceps, mm	10.3 ± 4.2	13.0 ± 4.7	17.8 ± 6.4	x
Subscapular, mm	7.6 ± 4.2	10.8 ± 4.7	16.4 ± 7.9	x
Sum of skinfold thickness	23.8 ± 10.1	30.5 ± 11.1	43.2 ± 18.7	x
Systolic blood press, mm Hg	102.3 ± 9.7	110.1 ± 10.8	113.5 ± 12.0	132.7 ± 15.4
Diastolic blood press, mm Hg	66.4 ± 7.4	71.4 ± 8.1	75.8 ± 10.7	81.7 ± 9.1
Total cholesterol, mg/dl	166.0 ± 24.4	163.3 ± 28.7	181.4 ± 35.6	234.7 ± 38.0
Triglycerides, mg/dl	67.5 ± 26.0	78.2 ± 32.6	84.6 ± 43.5	104.3 ± 43.5
HDL-Cholesterol, mg/dl	55.8 ± 8.4	54.9 ± 9.9	60.0 ± 13.9	66.1 ± 15.3
LDL-Cholesterol, mg/dl	96.6 ± 21.4	92.7 ± 22.7	104.7 ± 29.0	147.7 ± 32.4
NonHDL-Cholesterol, mg/dl	110.2 ± 22.8	108.3 ± 25.3	121.6 ± 30.9	168.6 ± 35.5
Total Cholesterol /HDL-C	3.0 ± 0.6	3.0 ± 0.6	3.1 ± 0.7	3.7 ± 0.9
LDL-C/HDL-C	1.8 ± 0.5	1.7 ± 0.5	1.8 ± 0.6	2.3 ± 0.7
TG/HDL-C	1.3 ± 0.7	1.5 ± 0.8	1.5 ± 1.0	1.7 ± 1.0
Apolipoprotein A-I, mg/dl	148.1 ± 16.5	150.3 ± 25.9	168.4 ± 35.2	181.6 ± 31.6
Apolipoprotein B, mg/dl	72.7 ± 15.0	73.4 ± 17.7	83.7 ± 20.6	109.6 ± 23.3
Glucose, mg/dl	81.0 ± 5.8	81.9 ± 9.7	83.8 ± 17.0	89.4 ± 10.5
Fasting insulin, μ/ml	7.7 ± 4.5	10.0 ± 5.0	7.7 ± 4.5	7.3 ± 4.8
HOMA-IR	1.6 ± 1.0	2.1 ± 1.3	1.6 ± 1.0	1.6 ± 1.2

**Table 2:** Prevalence (%) of risk factors in female participants

Age groups		3 – 11 years	12 – 18 years	19 – 55 years	> 55 years
		n = 141	n = 211	n = 151	n = 150
General overweight	(BMI>90^th^/ >25 kg/m^2^)	9.2	9.0	35.8	49.3
Central adiposity	(WC >90^th^ percentile/ 88 cm)	11.3	10.0	21.2	53.3
High LDL-C	(>130 mg/dl)	7.1	5.7	18.5	67.3
low HDL-C	(<50 mg/dl)	2.8	3.8	18.5	9.3
High Triglycerides	(≥ 150 mg/dl)	5.7	18.5	8.6	14.7
High SBP	(>95^th^ >130 mm Hg)	4.3	8.1	9.9	58.7
High DBP	(95^th^/ >85 mm Hg)	5.7	5.2	17.2	38.7

Women with median age of 14 years had the lowest fat intake and the highest consumption of carbohydrates whereas intake of protein was lowest in 10 year-old girls (Table 3). Relative (~36 energy %) and absolute (~70 g/day) fat consumption were similar in both groups of adults. The highest intake of saturated fat (~30 g/day) was seen in the 35 year-old women. Intake of unsaturated fat was lower in youths than in adults amounting to 37 g unsaturated fat respectively 53.4% of total fat consumption. The 68 year-old women had the highest intake of alcohol compared with young adults and adolescents and reported high consumption of fruit (226 g/day), vegetable (183 g/day) and whole grain bread (33 g/day). Daily intake of fiber increased continuously by age.

Overweight (OR 4.14; 95% CI 1.29-13.3) and central adiposity (5.9; 95% CI 1.6-22.5) were significantly associated with energy intake only in senior women. For all women, energy consumption was significantly associated with clustering of ≥3 cardiovascular risk factors (OR 4.72; 95% CI 1.22-18.33), low HDL-C (3.60; 95% CI 1.41-9.20) and high non-HDL-C (2.74; 95% CI 1.38-5.45). However, we did not find any association between dietary fat consumption and CVD risk factors in any age group of women.

**Table 3:** Daily consumption of macronutrients (mean ± SD) in female participants

Age, groups	3 – 11 years	12 – 18 years	19 – 55 years	> 55 years
	n = 50	n = 57	n = 40	n = 138
Energy, kcal	1566.1 ± 272.4	1728.2 ± 451.4	1790.2 ± 448.7	1715.8 ± 383.2
Fat, % energy	35.1 ± 4.4	32.7 ± 6.0	35.6 ± 6.1	36.2 ± 5.5
Carbohydrates, % energy	51.2 ± 5.1	52.3 ± 6.7	47.1 ± 7.9	45.7 ± 6.5
Protein, % energy	13.6 ± 2.2	14.5 ± 2.2	15.9 ± 4.3	15.7 ± 2.8
Fat, g	61.1 ± 13.7	63.4 ± 20.6	70.8 ± 22.5	69.7 ± 22.2
SAFA, g	26.8 ± 7.2	27.3 ± 9.8	30.1 ± 11.1	27.6 ±10.2
MUFA, g	21.3 ± 5.0	21.5 ± 7.4	24.5 ± 8.0	25.0 ± 8.6
PUFA, g	8.7 ± 2.1	10.1 ± 3.5	11.2 ± 3.9	12.3 ± 4.5
Cholesterol, mg	217.3 ± 63.0	239.1 ± 108.3	250.1 ± 96.7	242.3 ± 104.6
Carbohydrates, g	197.0 ± 38.7	221.0 ± 62.6	207.5 ± 60.3	190.9 ± 44.0
Monosaccharide, g	34.0 ± 13.1	42.3 ± 24.9	37.7 ± 16.6	37.0 ± 14.6
Disaccharide, g	55.2 ± 19.0	59.7 ± 27.7	61.1 ± 25.7	54.3 ± 21.2
Polysaccharides, g	105.6 ± 24.1	116.9 ± 34.1	106.6 ± 32.8	96.2 ± 24.2
Saccharose, g	44.9 ± 15.4	49.1 ± 25.8	50.9 ± 22.6	43.7 ± 17.7
Fiber, g	15.5 ± 4.1	17.3 ± 6.2	19.3 ± 6.6	21.7 ± 6.0
Protein, g	52.2 ± 11.6	60.7 ± 15.2	67.6 ± 17.8	65.8 ± 17.5
Plant Protein, g	22.0 ± 4.8	25.0 ± 7.8	25.2 ± 7.1	24.7 ± 6.0
Water, ml	1445.7 ± 315.4	2067.6 ± 1117.0	2554.5 ± 805.4	2512.3 ± 737.5
Alcohol, g	0.3 ± 0.3	0.9 ± 2.9	4.2 ± 5.8	6.0 ± 7.4
Alcohol, % energy	0.0 ±0.1	0.3 ± 1.0	1.6 ± 2.3	2.3 ± 2.9

## DISCUSSION

This cross-sectional community-based study demonstrates that the prevalence of CVD risk factors increases continuously in women from childhood to senior age. Consumption of carbohydrates was higher in youths than in adults but daily intake of fiber increased continuously by age but was far less than 30-35 g/day recommended by heart health fare.^20^

In 6-7 year-old Spanish children from 4 cities with slightly lower mean age and lower BMI, the intakes of Kcal/day, fat and protein were higher while the carbohydrate consumption was considerably lower (37-39 energy %) compared to our study.^21^ The DISC Study described that 9.5 ± 0.7 year-old US children consuming 33.4% fat and 12.5% saturated fat at baseline had total cholesterol of 200 mg/dl, LDL-C of 130 mg/dl, triglycerides of 334 mg/dl and HDL-C of 57 mg/dl^22^ which were considerably higher than those in our study. Comparison with two studies demonstrates that senior participants of the multiethnic Women’s Health Initiative^9^ were slightly younger (61.8± 6.9 vs. 68.6 ± 4-8 years) but had a more adverse lipid profile, higher SBP and higher BMI than women participating in the PEP Family Heart Study. The Nurses’ Health Study found that the inverse association between polyunsaturated fat intake and CHD risk was stronger in overweight (BMI ≥ 25 kg/m^2^) and in younger (≤ 65 years) women.^23^ This might correspond to our findings that the association between energy intake and overweight was considerably stronger in the younger adults compared with the senior group; however, we did not find any significant association between fat intake and overweight. Also, the EPIC Study did not find any association between weight gain and the amount and the type of fat in women of comparable age and BMI consuming a similar diet (35 % energy fat, 45% energy carbohydrates, and 17% of energy protein).^24^ Thirty six percent of energy intake of fat in this study was higher than that recommended for adults by the heart-health fare diet^20^ and the central European recommendations^19^ whereas 47% of energy consumption of carbohydrates was lower than that recommended (55% of energy) by these diets. Sixteen percent of energy protein intake corresponds to that of the European recommendations while the American advice 18-25% of energy protein intake. As demonstrated previously, a diet providing 37% of energy by fat with a P/S ratio (polyunsaturated to saturated fatty acids) of 1 decreased LDL-C without affecting HDL, the subfractions HDL_2_ and HDL_3_ and apolipoproteins A-I and A-II.^25^

Though 1-year intervention did not improve the adverse lipid profile of the participants in the FIT Heart Study, the dietary score did improve significantly in the intervention group (18.4%) compared with the control group (5.0%). This is explained by the specific information and guidance in the intervention group of which 50% were unaware of their elevated blood pressure and increased LDL- C.^6^ This suggestion is supported by our experience demonstrating that regular control of risk factors was the first step of successful intervention and that awareness of risk is easily obtained by simple and inexpensive measurements.^13^,^15^ This is strongly recom-mended as one way to implement the current American Heart Association diet and lifestyle recommendations in children and adults.^25^,^26^

In summary, the CVD risk profile was lower in girls and adolescents than in the age group of 19-55 years but, steeply increased beyond age 55 reaching prevalence rates above 50% for adiposity, hypertension and LDL-Cholesterol. Caloric intake was not different in adolescents and adults, but consumption of carbohydrates decreased and intake of fat and proteins increased with age among adults compared with youths. The daily intake of fiber, polyunsaturated and monounsaturated fats increased continuously with increasing age. While fat consumption was not associated with CVD risk factors, we found a significant association of energy intake with low HDL-C, high non-HDL-C and risk factor clustering in the whole sample of female participants. The strength of this crosssectional monoethnic study lay in the inclusion of three generations of women participating in this community-based family study with yearly controls of CVD risk factors and 7-day dietary records. Limitations are the missing information on the association between diet and CVD events and the lack of comparisons between questionnaires and weighted food records. Another limitation is that confounding factors like physical activity were not evaluated in this study.

## CONCLUSIONS

From children to senior women, the prevalence of CVD risk factors increased continuously resulting in increasing prevalence rates for systolic hypertension, increased LDL-cholesterol, and general and central adiposity of above 50– 60% compared with the prevalence rates of about 10% in children and adolescents. Consumption of protein and fiber increased continuously over the age groups whereas carbohydrate intake decreased and total fat consumption was similar in children and adults while adolescents had a 3% lower energy intake. There was no significant association between fat intake and CVD risk factors. However, we found a very strong association between energy intake and adiposity in adults but not in youths. Prospective studies in larger samples of both genders including confounding influences like physical activity are needed.
